# Point-of-care Ultrasound to Distinguish Retinal Detachment and Ruptured Arterial Microaneurysm

**DOI:** 10.5811/cpcem2022.4.55301

**Published:** 2022-08-12

**Authors:** Rachel Kester, Scott Szymanski, Michael Perreault

**Affiliations:** Madigan Army Medical Center, Department of Emergency Medicine, Joint Base Lewis-McChord, Washington

**Keywords:** point-of-care ultrasound, preretinal hemorrhage, retinal detachment

## Abstract

**Case presentation:**

We present the case of an older male with point-of-care-ultrasound (POCUS) imaging consistent with retinal detachment who was instead found by ophthalmology to have a ruptured arterial microaneurysm with vitreous and preretinal hemorrhage. The patient later had complete resolution of his symptoms. We discuss this retinal detachment “mimic.”

**Discussion:**

Preretinal hemorrhage is an uncommon condition that can be mistaken for ophthalmologic emergencies such as retinal detachment. The images and videos shown here add to the body of evidence that POCUS is useful in diagnosing pre-retinal hemorrhage but must be differ-entiated from retinal detachment. These images also emphasize the need for further research and application of POCUS for the identification of preretinal hemorrhage.

## CASE PRESENTATION

A 64-year-old male presented to the emergency department (ED) with sudden, dark, “curtain-like” painless vision loss in his right eye after heavy lifting three days earlier. Vision loss had since resolved; however, the patient complained of continued persistent floaters and blurriness. Visual acuity in his left eye was 20/20 and 20/60 in his right eye without diplopia. He had intact extraocular movements with pupils equal and reactive to light. Intraocular pressure was 13 millimeters of mercury in both eyes. Point-of-care ultrasound (POCUS) findings were concerning for a retinal detachment and vitreous hemorrhage (Video). The patient was not on any blood thinner medication. No further workup was obtained in the ED and ophthalmology consult determined the final diagnosis to be a ruptured arterial microaneurysm at the superotemporal arcade with vitreous and preretinal hemorrhage.

## DISCUSSION

Valsalva retinopathy is an uncommon condition most typically seen in young males following sudden increases in intra-abdominal pressure from activities such as vomiting or, as in the case of our patient, weightlifting, which cause a spontaneous rupture of ocular capillaries.^1^ While this condition is mostly self-limited with a favorable visual prognosis, it is imperative to distinguish this condition from other ophthal-mologic emergencies that require immediate intervention, such as a retinal detachment. Diagnosing retinal detachment via POCUS has been shown to have a sensitivity of 97% and specificity of 88%.^2^ Ultrasound findings of a bright, continuous, folded membrane with independent excursion upon recruitment of extraocular muscles while visualizing the optic nerve are highly suggestive of a retinal detachment but cannot rule out a ruptured arterial microaneurysm based on POCUS alone.^2^

Thus, these ultrasound findings warrant an ophthalmologic consult for definitive diagnosis and treatment. While this is not a diagnosis typically made by an emergency physician, it is important for the emergency physician to be aware of such retinal detachment “mimics” when discussing with consultants and patients, especially when valsalva is involved in the history. In these cases, however, retinal detachment remains the “must not miss” diagnosis.

**CPC-EM Capsule**

What do we already know about this clinical entity?

*We know about the treatment and management of preretinal hemorrhage and retinal detachment but not how the two entities differ diagnostically*.

What is the major impact of the images?

*Preretinal hemorrhage appears similar to retinal detachment on orbital ultrasound*.

How might this improve emergency medicine practice?

*Point-of-care-ultrasound can be used to diagnose preretinal hemorrhage, while demonstrating that ophthalmologic emergencies such as retinal detachment cannot be ruled out*.

## Supplementary Information

Video Point-of-care ultrasound on patient’s right eye demonstrating a bright, echogenic undulating membrane extending across the posterior vitreous area that is highly concerning for retinal detachment.
